# Linkage Relationships Among Multiple QTL for Horticultural Traits and Late Blight (*P. infestans*) Resistance on Chromosome 5 Introgressed from Wild Tomato *Solanum habrochaites*

**DOI:** 10.1534/g3.113.007195

**Published:** 2013-10-11

**Authors:** J. Erron Haggard, Emily B. Johnson, Dina A. St. Clair

**Affiliations:** Plant Sciences Department, University of California-Davis, Davis, California 95616

**Keywords:** tomato, *Solanum lycopersicum*, introgression, QTL mapping, linkage drag

## Abstract

When the allele of a wild species at a quantitative trait locus (QTL) conferring a desirable trait is introduced into cultivated species, undesirable effects on other traits may occur. These negative phenotypic effects may result from the presence of wild alleles at other closely linked loci that are transferred along with the desired QTL allele (*i.e.*, linkage drag) and/or from pleiotropic effects of the desired allele. Previously, a QTL for resistance to *Phytophthora infestans* on chromosome 5 of *Solanum habrochaites* was mapped and introgressed into cultivated tomato (*S. lycopersicum*). Near-isogenic lines (NILs) were generated and used for fine-mapping of this resistance QTL, which revealed coincident or linked QTL with undesirable effects on yield, maturity, fruit size, and plant architecture traits. Subsequent higher-resolution mapping with chromosome 5 sub-NILs revealed the presence of multiple *P. infestans* resistance QTL within this 12.3 cM region. In our present study, these sub-NILs were also evaluated for 17 horticultural traits, including yield, maturity, fruit size and shape, fruit quality, and plant architecture traits in replicated field experiments over the course of two years. Each previously detected single horticultural trait QTL fractionated into two or more QTL. A total of 41 QTL were detected across all traits, with ∼30% exhibiting significant QTL × environment interactions. Colocation of QTL for multiple traits suggests either pleiotropy or tightly linked genes control these traits. The complex genetic architecture of horticultural and *P. infestans* resistance trait QTL within this *S. habrochaites* region of chromosome 5 presents challenges and opportunities for breeding efforts in cultivated tomato.

The value of wild species as sources of genetic diversity for breeding their crop species relatives has long been appreciated, and there are many examples of their use in crop improvement (Labate and Robertson 2012). In modern breeding, introgression of genes controlling useful traits from wild into cultivated species is often facilitated by first associating molecular marker genotypes with trait phenotypes, a process known as gene mapping or quantitative trait locus (QTL) mapping (Collard *et al.* 2005). Once the chromosomal locations of QTL controlling a particular trait are known, marker-assisted selection (MAS) can be used to transfer wild alleles with favorable trait effects into a crop species while simultaneously selecting against wild alleles at other loci to create improved varieties (Xu and Crouch 2008). MAS breeding can be particularly effective in the improvement of quantitative traits, traits that are controlled by a few to many genes that may interact with each other and with the environment (St. Clair 2010). However, effective use of QTL alleles from wild species can be complicated by linkages among desirable and undesirable trait loci and/or by interactions of QTL with the environment (Bernardo 2008). Genes controlling a desirable trait may also affect one or more other traits, a phenomenon known as pleiotropy (Chen and Luebberstedt 2010).

Tomato is an important crop worldwide and is the second most valuable vegetable in United States production (National Agriculture Statistics Service 2011). Cultivated tomato (*Solanum lycopersicum*) has limited genetic diversity in comparison with its diverse wild tomato species relatives, primarily because of genetic bottlenecks during domestication (Miller and Tanksley 1990; Park *et al.* 2004). Wild *Solanum habrochaites* is an important source of genetic diversity for tomato improvement. Among the traits that can be improved via introgressions from *S. habrochaites* are horticultural traits, such as yield, fruit size, and fruit quality (Bernacchi *et al.* 1998b; Monforte and Tanksley 2000; Yates *et al.* 2004; Ben Chaim *et al.* 2006; Mathieu *et al.* 2009), and resistance to diseases, such as late blight, bacterial canker, gray mold, and early blight (Foolad *et al.* 2002; Zhang *et al.* 2003; Brouwer *et al.* 2004; Brouwer and St. Clair 2004; Coaker and Francis 2004; Finkers *et al.* 2007). Most of these horticultural and resistance traits are quantitatively inherited.

A very desirable trait of *S. habrochaites* is its resistance to *Phytophthora infestans*, the oomycete pathogen responsible for late blight disease of tomato and its close relative, potato (Brouwer *et al.* 2004; Foolad *et al.* 2008). Late blight disease causes significant losses in tomato, resulting in approximately $5 billion in crop losses and chemical control costs annually (Judelson and Blanco 2005). Brouwer *et al.* (2004) mapped QTL for quantitative resistance to *P. infestans* from *S. habrochaites* on each of the 12 tomato chromosomes. Brouwer and St. Clair (2004) further refined the locations of three of the QTL (on chromosomes 4, 5, and 11) by fine-mapping with near-isogenic lines (NILs). These three resistance QTL were also found to be colocated and/or linked with some QTL having negative effects on horticultural traits, such as plant height, plant shape, maturity, yield, and fruit size, suggesting linkage drag.

Our interests in understanding the genetic basis of QTL controlling horticultural traits and their linkage relationships with QTL for resistance to *P. infestans*, as well as in developing material useful for breeding improved tomato varieties, led us to conduct further investigations regarding the introgressed chromosome 5 region from *S. habrochaites*. In a companion study (Johnson *et al.* 2012), we mapped the chromosome 5 resistance QTL reported by Brouwer and St. Clair (2004) at higher resolution with sub-NILs. We found that the resistance QTL located in a 12.3 cM region subsequently fractionated into two and three QTL groups for foliar and stem resistance, respectively. In the present study we used the same set of chromosome 5 sub-NILs as Johnson *et al.* (2012), focusing on mapping loci associated with horticultural traits and determining linkage relationships among these loci and the late blight resistance QTL. Our goals were as follows: (1) to determine the genetic architecture and environmental stability of QTL controlling horticultural traits within the chromosome 5 introgressed region from *S. habrochaites*; (2) to determine the linkage relationships among loci controlling horticultural and *P. infestans* resistance traits; and (3) to assess the implications of trait genetic architecture, QTL environmental stability, and linkage relationships among QTL for the potential improvement of cultivated tomato via breeding.

## Materials and Methods

### Plant materials, genotyping, and marker-assisted selection

We developed a set of sub-NILs in *S. lycopersicum* for a chromosome 5 introgression from a *P. infestans*–resistant donor parent, wild tomato *S. habrochaites* accession LA 2099, via marker-assisted selection during backcrossing and selfing generations, as described by Johnson *et al.* (2012). Genomic DNA extractions, genotyping with chromosome 5 PCR-based markers (SCAR, CAPS, and SSR), primer sequences, enzymatic reaction conditions, and restriction enzymes used for each marker have been described by Johnson *et al.* (2012).

We genotyped 1589 BC_6_S_1_ progeny to identify recombinant sub-NIL progeny; a subset of 652 progeny (150 recombinant plus 502 nonrecombinant) was used to construct a linkage map for the introgressed region (see *Linkage and QTL mapping* section). Heterozygous recombinant BC_6_S_1_ individuals underwent self-pollination and progeny were marker-selected to obtain homozygous recombinant BC_6_S_2_ sub-NILs. These plants underwent self-pollination to obtain ample BC_6_S_3_ sub-NIL seeds for replicated field experiments. We evaluated 58 BC_6_S_3_ sub-NILs in the 2009 field experiments. In the 2010 field experiments, a subset of 41 of the 58 sub-NILs was evaluated to allow increased replication per location while reducing genetic redundancy, as explained by Johnson *et al.* (2012). Graphical marker genotypes for the 58 selected BC_6_S_3_ sub-NILs are presented in Supporting Information, Table S1.

### Field experimental design and procedures

BC_6_S_3_ sub-NILs and the parental NIL from which they were derived (subsequently referred to as NIL5) were evaluated in replicated experiments at field locations in Salinas, California (designated as locations 1 and 2) and in Davis, California (locations 3 and 4) over the course of 2 years. Summer in Salinas is generally cool and humid, which is conducive to late blight disease development, whereas Davis summers are warm and dry, typical of processing tomato production areas in California’s Central Valley.

Seedlings were grown in a greenhouse for 6 wk and then transplanted into the field locations. Sixty-one genotypes (NIL5, 58 sub-NILs for chromosome 5, and two commercial processing cultivars, E6203 and Hypeel 45) and 44 genotypes (NIL5, 41 sub-NILs, Hypeel 45, and E6203) were included in the 2009 and 2010 experiments, respectively. Experiments were arranged in a randomized complete block design. For both years, one plot per genotype per block was included, except for controls, for which there were two plots per block. In 2009, three blocks per location were used. In 2010, use of a reduced number of 41 sub-NILs enabled replication to be increased to five blocks in each of locations 1 and 2 and to four blocks in each of locations 3 and 4. At each of the four locations, each plot consisted of five plants spaced 0.30 m apart in rows separated by 1.02 m in locations 1 and 2, and by 1.52 m in locations 3 and 4. Border rows and plots with the cultivar E6203 surrounded each experiment at each location. Standard horticultural field practices for processing tomato were used at all locations. Locations 1 and 2 were sprinkler-irrigated, whereas locations 3 and 4 were furrow-irrigated, as needed.

### Phenotypic trait evaluations

All traits (Table 1) were evaluated on a per-plot basis. Vegetative horticultural traits were evaluated at all four locations. Late blight disease was only evaluated in Salinas (locations 1 and 2) because this disease did not occur in Davis, as expected, because of typical warm, dry summers. Reproductive traits were only evaluated at Davis (locations 3 and 4) because of logistics of timely sampling of ripe fruit. Vegetative horticultural traits evaluated were plant height (H) and width (W) in cm, canopy density (CD; visual rating scale: 1 = very sparse to 5 = very dense), and plant habit (HAB; visual rating scale: 1 = prostrate to 5 = very upright). H, W, CD, and HAB were assessed at locations 1 and 2 at 71 and 46 days after planting (DAP) in 2009 and 2010, respectively. At locations 3 and 4, these traits were evaluated at 80 DAP in 2009 and at 68 (H and W) and 73 (CD and HAB) DAP in 2010. From plant height and width, two secondary traits were derived, plant size (SZ; product of height × width) and shape (SH; ratio of height to width). The reproductive horticultural traits measured or scored were as follows. DAP to maturity was evaluated at two stages of maturity: when each plant in the plot had its first ripe fruit (DAP1st) and when 50% of fruit in a plot were ripe (DAP50). The weight of 30 ripe fruits was evaluated when 50% of fruit in a plot were ripe (30Wt). Yield in kg (YLD) was evaluated when 95% of the fruit in a plot were ripe. Ripe fruit were used to obtain the weight of 100 seeds (SW), which was measured only in 2009 because of labor limitations. The ripe fruit quality traits pH and Brix (*i.e.*, sugar content or soluble solids) were measured using a pureed sample of 10 whole fruits obtained from plots with 50% ripe fruit using an Oakton pH Testr2 (Oakton Instruments, Vernon Hills, IL) and a Reichert AR200 digital refractometer (Reichert Technologies, Buffalo, NY), respectively. Size traits obtained for ripe fruit were fruit perimeter (FP), fruit width (FW; width at mid height), and fruit height (FH; height at mid width). These traits were measured on flatbed scanner images of eight longitudinally sliced fruit per plot using Tomato Analyzer software (Brewer *et al.* 2006), which refers to fruit length as height and to fruit longitudinal circumference as perimeter. From FH and FW, the secondary variable, fruit shape (FS; ratio of FH to FW), was obtained. Trait names, abbreviations, and brief descriptions are given in Table 1.

**Table 1:** Abbreviations for traits evaluated in this study

Trait Type	Abbreviation	Description
Late blight	LEAF	AUDPC for foliar symptoms
	STEM	AUDPC for stem symptoms
Maturity	DAP1st	Time after planting to first ripe fruit (d)
	DAP50	Time after planting to 50% ripe fruit (d)
Yield	YLD	Fruit yield (kg)
Fruit size/shape	FH	Fruit height (mm)
	FW	Fruit width (mm)
	FS	Fruit shape (FH × FW; mm^2^)
	FP	Fruit perimeter (mm)
	30Wt	Weight of 30 fruits (g)
Fruit quality	Brix	Brix (soluble solids content)
	pH	Fruit acidity
Plant	CD	Canopy density (visual rating: 1 = very sparse to 5 = very dense)
architecture	HAB	Plant habit (visual rating: 1 = prostrate to 5 = very upright)
	H	Plant height (cm)
	W	Plant width (cm)
	SH	Plant shape (H:W; cm^2^)
	SZ	Plant size (H × W; cm^2^)
	SW	Weight of 100 seeds (g)

AUDPC, area under the disease progress curve.

On September 15, 2009, Salinas locations 1 and 2 were inoculated with a local *P. infestans* isolate per Johnson *et al.* (2012). In 2010 in mid September, a natural *P. infestans* infection occurred in both locations, precluding the need for inoculation. As described by Johnson *et al.* (2012), phenotypic scoring of late blight disease symptoms was performed visually and symptom data were used to calculate area under the disease pressure curve (AUDPC) for foliar and stem disease symptom progression (referred to as LEAF and STEM, respectively). Lower AUDPC values indicate less disease symptom progress and are therefore indicative of increased disease resistance.

### Statistical data analysis

Data for each trait (Table S2) were tested for normality using the Shapiro/Wilk W statistic in PROC UNIVARIATE in SAS (SAS Institute, Cary, NC) and for homogeneity of variance using the Levene's test. Data for heteroscedastic traits were weighted by the reciprocal of the variance for those terms with significant departure from the assumption of equal variance. ANOVA for each trait was performed using PROC GLM in SAS with the following linear additive model for a randomized complete block design and multiple locations:

Trait=Loc+Block(Loc)+Genotype+Loc*Genotype

where Trait was a given phenotypic trait, Loc was the effect of location, Genotype was the individual sub-NIL or control (NIL5, E6203, Hypeel 45), * indicated an interaction, and parentheses indicated a nested variable. Block(Loc) was considered a random variable. Significant genotype × location interactions were detected in 2009 for traits LEAF, DAP50, YLD, FH, FS, FP, 30Wt, Brix, pH, CD, H, W, SH, and SZ, and in 2010 for STEM, DAP1st, DAP50, FH, FW, FS, FP, 30Wt, Brix, pH, height, width, SH, and SZ. For these traits, separate analyses were conducted for each location. PROC MIXED in SAS was used to estimate least squares means, because of missing data for some traits, and to perform means separation with Tukey's HSD.

Pearson correlation coefficients (*r*) were calculated for pairwise combinations of all trait genotypic means in 2009 and in 2010 using Proc CORR in SAS. Only significant (*P* ≤ 0.05) correlations ≥0.4 are reported.

### Linkage and QTL mapping

A linkage map for the introgressed region was constructed using DNA marker genotype data across 17 loci for 652 BC_6_S_1_ progeny (150 recombinants plus 502 nonrecombinants). The map was constructed with JoinMap 3.0 (van Ooijen and Voorips 2001) using the Kosambi mapping function and a 3-LOD significance threshold. After the release of the tomato genome sequence (Sato *et al.* 2012), we developed additional markers using the SL2.40 genome build (http://solgenomics.net) to help define the physical extent of the chromosome 5 *S. habrochaites* introgression within NIL5 (File S1) .

The Composite Interval Mapping (CIM) module in WinQTLCartographer2.5 (Wang *et al.* 2011) was used for detection of QTL using sub-NIL means obtained from ANOVA for each trait. QTL mapping was performed using CIM Model 6 (standard model) and the forward and backward regression method with a walk speed of 1 cM and a window size of 2 cM. Trait-specific permuted LOD thresholds (*P* = 0.05) were empirically established for each trait using 1000 permutations (Churchill and Doerge 1994) in WinQTLCartographer.

A QTL for a trait was considered significant at *P* ≤ 0.05 if the peak LOD value exceeded the trait-specific permuted threshold. Multiple QTL were declared for a single trait when the LOD values between significant (*P* ≤ 0.05) peaks within the introgressed region decreased below the significance threshold for at least two contiguous markers. Each significant QTL was denoted by trait name, location, and year. For example, DAP1st34_2009 is a QTL detected in the analysis of DAP1st trait data from locations 3 and 4 in 2009.

**Figure 1:**
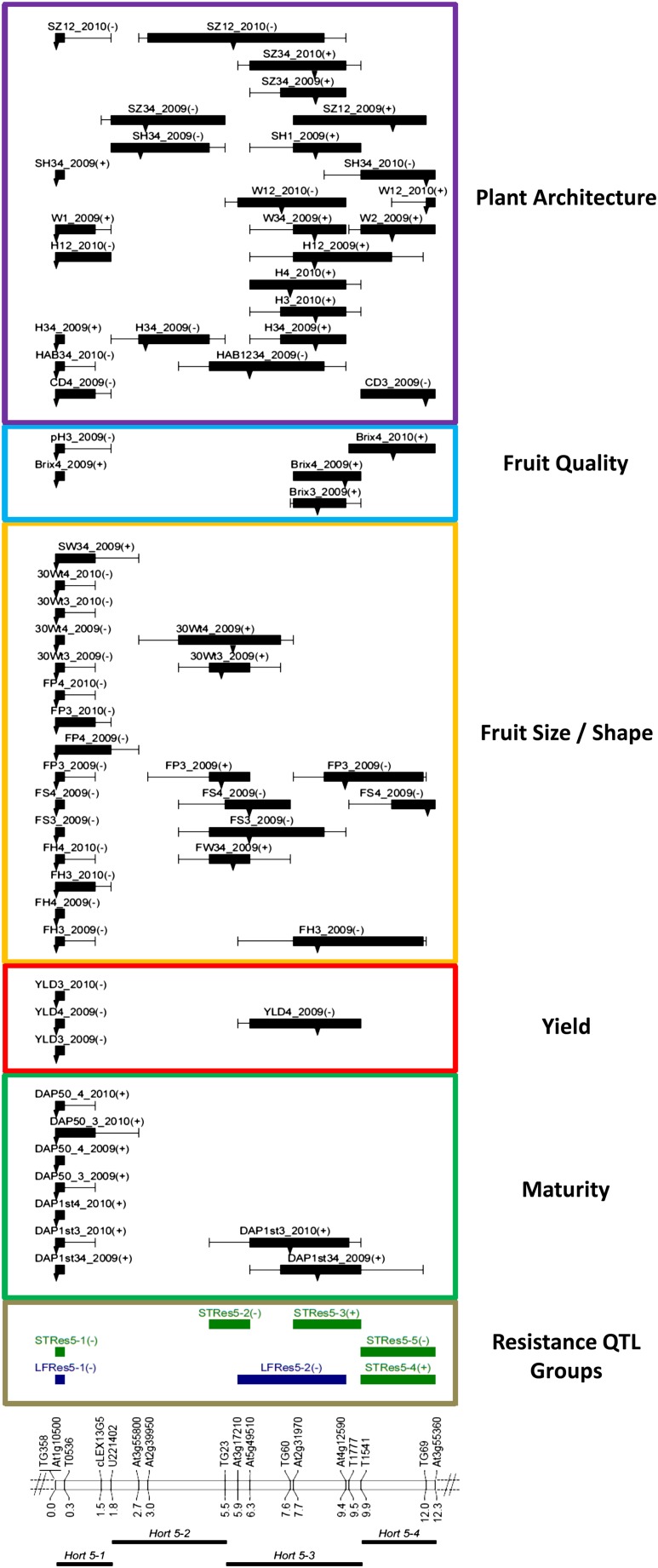
QTL mapped to a chromosome 5 region introgressed from *Solanum habrochaites* to *S. lycopersicum*. Horticultural trait QTL and *Phytophthora infestans* resistance QTL groups detected in chromosome 5 sub-NILs evaluated in 2009 and 2010 field experiments, sorted by trait class. Below the linkage map are horticultural trait QTL group names, locations, and distances in cM; above the linkage map are *P. infestans* resistance trait QTL groups (*LfRes* and *StRes* refer to LEAF and STEM resistance, respectively) (Johnson *et al.* 2012) and QTL detected for horticultural traits, sorted by trait class. Boxes and whiskers show 1-LOD and 2-LOD intervals, respectively. Arrows on QTL bars indicate LOD peak locations. QTL names are given by trait, location(s), and year evaluated (see *Materials and Methods* section). The effect of the *S. habrochaites* allele at a QTL is indicated after the QTL name: (−) indicates a decrease in that trait value.

A linkage map showing locations of significant QTL was constructed using MapChart2.1 (Voorips 2002). QTL locations were indicated as 1-LOD bars and 2-LOD whiskers (Figure 1). For purposes of discussion, QTL were assigned to QTL trait groups (delineated as *Hort 5-1* through *Hort 5-4*) based on coincidence of their 1-LOD support intervals. Although a few of the QTL have 1-LOD support intervals that extended beyond the boundary of their assigned group, their peak locations supported their placement in their respective groups.

Comparisons were made among QTL for *P. infestans* resistance traits (LEAF and STEM) (Johnson *et al.* 2012) and horticultural traits for QTL coincidence by visual inspection of their chromosomal locations on the linkage map. A statistical test based on the hypergeometric probability distribution (Lin *et al.* 1995) was used to calculate QTL correspondence, the probability of obtaining the observed number of matching QTL by chance. A QTL match was declared when the 1-LOD support intervals overlapped. The number of comparison intervals was seven, based on the average size of our resistance and horticultural trait QTL (1.9 cM) and the overall map distance of the *S. habrochaites* introgression (12.3 cM).

Our QTL locations were also compared to those previously reported for both disease resistance and horticultural trait QTL on chromosome 5 in tomato and in potato, based on common markers as well as genomic sequence data for both crop species. Sources used for QTL location comparisons included the following: tomato (Eshed and Zamir 1996; Bernacchi *et al.* 1998b; Zhang *et al.* 2002; Causse *et al.* 2004; Coaker and Francis 2004; Semel *et al.* 2006; Brewer *et al.* 2007; Jones *et al.* 2007); potato (Collins *et al.* 1999; Visker *et al.* 2003; Visker *et al.* 2005; Sliwka *et al.* 2007; Achenbach *et al.* 2009; Danan *et al.* 2011); and genomic sequences (http://solgenomics.net; Xu *et al.* 2011; Sato *et al.* 2012). When common markers were not available, the Tomato-Expen 2000 map (Fulton *et al.* 2002b) available on the Sol Genomics Network (http://solgenomics.net; Bombarely *et al.* 2011) was used to facilitate map alignment.

### Selection of sub-NIL breeding lines

Truncation selection was applied sequentially for traits LEAF, YLD, FP, 30Wt, and DAP1st to identify breeding lines potentially useful for development of tomato varieties with improved resistance to *P. infestans*. Out of 41 sub-NILs evaluated in both years, the first round of truncation removed seven lines with LEAF values less resistant than control cultivar E6203 in 2 years or locations. The second round removed eight lines with YLD <66% of E6203 in 2 years or locations, whereas the third round removed six lines with FP <92% of E6203 in 2 years or locations. The fourth round removed six lines with 30Wt <80% of E6203 in 2 years or locations. The final round removed three lines with DAP1st more than 10 days later than E6203 in 2 years or locations; however, five lines that would have been removed based on this criterion were kept because of their relatively higher levels of foliar resistance to *P. infestans* (*i.e.*, lower LEAF values). At the end of the selection process, 11 lines were chosen.

## Results

### ANOVA

In 2009, 61 genotypes (sub-NILs and controls) were evaluated for late blight disease symptom traits (Johnson *et al.* 2012) and horticultural traits (Table 1). For all traits, genotypes were significantly different (*P* ≤ 0.05) (Table 2). Significant genotype × location interactions were detected in 2009 for LEAF, DAP50, YLD, FH, FS, FP, 30Wt, Brix, pH, CD, H, W, SH, and SZ. As a result, these traits were analyzed separately by location. *R*^2^ values per trait ranged from 0.45 to 0.91.

**Table 2:** Summary of ANOVA performed on trait data

					F Values		
Trait Class	Trait Code	Trait	Year	Location	Genotype	Location	*R*^2^
Late blight	LEAF	Leaf AUDPC	2009	1	1.69^‡^	—	0.47
resistance				2	2.77^‡^	—	0.77
			2010	1 & 2	7.02^‡^	68.70^‡^	0.77
	STEM	Stem AUDPC	2009	1 & 2	5.03^‡^	0.01 ns	0.64
			2010	1	5.63^‡^	—	0.59
				2	5.78^‡^	—	0.66
Maturity	DAP1st	Time to first ripe fruit (d)	2009	3 & 4	11.01^‡^	44.19^†^	0.78
			2010	3	14.94^‡^	—	0.83
				4	7.13^‡^	—	0.71
	DAP50	Time to 50% ripe fruit (d)	2009	3	12.82^‡^	—	0.86
				4	6.88^‡^	—	0.77
			2010	3	34.47^‡^	—	0.92
				4	39.79^‡^	—	0.86
Yield	YLD	Yield	2009	3	6.25^‡^	—	0.76
				4	2.74^‡^	—	0.60
			2010	3	3.89^‡^	—	0.56
Fruit size/shape	FH	Fruit height	2009	3	6.37^‡^	—	0.77
				4	10.13^‡^	—	0.83
			2010	3	19.30^‡^	—	0.86
				4	14.53^‡^	—	0.83
	FW	Fruit width	2009	3 & 4	5.07^‡^	26.66^†^	0.68
			2010	3	5.89^‡^	—	0.66
				4	7.35^‡^	—	0.71
	FS	Fruit shape	2009	3	18.79^‡^	—	0.91
				4	18.26^‡^	—	0.90
			2010	3	23.99^‡^	—	0.89
				4	16.88^‡^	—	0.85
	FP	Fruit size	2009	3	3.14^‡^	—	0.63
				4	3.73^‡^	—	0.65
			2010	3	8.44^‡^	—	0.74
				4	9.35^‡^	—	0.76
	30Wt	Fruit weight	2009	3	11.03^‡^	—	0.84
				4	6.97^‡^	—	0.77
			2010	3	14.84^‡^	—	0.83
				4	14.65^‡^	—	0.83
	SW	Seed weight	2009	3 & 4	7.45^‡^	27.11^‡^	0.71
Fruit quality	Brix	Brix	2009	3	4.50^‡^	—	0.69
				4	4.44^‡^	—	0.71
			2010	3	10.79^‡^	—	0.78
				4	6.39^‡^	—	0.69
	pH	pH	2009	3	2.21^‡^	—	0.51
				4	2.71^‡^	—	0.58
			2010	3	2.83^‡^	—	0.49
				4	2.44^‡^	—	0.45
Plant architecture	CD	Canopy density	2009	1 & 2	4.97^‡^	0.32 ns	0.59
				3	2.78^‡^	—	0.59
				4	5.48^‡^	—	0.72
			2010	3 & 4	4.66^‡^	44.15^‡^	0.58
	H	Plant height	2009	1 & 2	8.34^‡^	0.30 ns	0.69
				3 & 4	9.75^‡^	60.27†	0.76
			2010	1 & 2	5.75^‡^	426.88‡	0.67
				3	5.95^‡^	—	0.66
				4	7.55^‡^	—	0.52
	W	Plant width	2009	1	3.85^‡^	—	0.71
				2	2.54^‡^	—	0.55
				3 & 4	6.27^‡^	252.89	0.72
			2010	1 & 2	4.71^‡^	75.32^‡^	0.54
				3 & 4	15.92^‡^	26.86^†^	0.75
Plant architecture	SH	Plant shape (H:W)	2009	1	1.60*	—	0.54
				2	1.75^†^	—	0.48
				3 & 4	3.91^‡^	3.19 ns	0.55
			2010	1 & 2	3.81^‡^	106.51^‡^	0.43
				3 & 4	7.29^‡^	81.65^‡^	0.61
	SZ	Plant size	2009	1 & 2	9.06^‡^	0.31 ns	0.72
				3 & 4	11.24^‡^	193.22^‡^	0.80
			2010	1 & 2	5.60‡	192.19^‡^	0.66
				3 & 4	17.28^‡^	4.01 ns	0.75
	HAB	Plant habit	2009	1, 2, 3, 4	2.83^‡^	38.50^‡^	0.45
			2010	3 & 4	7.57^‡^	11.42*	0.59

F test values and *R*^2^ values are presented for each analysis by trait, year, and location or combination of locations (see *Materials and Methods* section). *R*^2^ indicates the fit of the data to the linear additive model for each analysis. Late blight resistance results are from Johnson *et al.* (2012). —, not included in model; AUDPC, area under the disease progress curve; ns, not significant.

* *P* ≤ 0.05.

† *P* ≤ 0.01.

‡ *P* ≤ 0.001.

In 2010, 41 genotypes were evaluated for disease symptom traits and horticultural traits (Table 1). For all traits, genotypes were significantly different (*P* ≤ 0.05) (Table 2). Significant genotype × location interactions were detected in 2010 for STEM, DAP1st, DAP50, FH, FW, FS, FP, 30Wt, Brix, pH, H, W, SH, and SZ. Therefore, these traits were analyzed separately by location. *R*^2^ values per trait ranged from 0.43 to 0.89. In general, foliar resistance to *P. infestans* (LEAF) exhibited higher *R*^2^ values than stem resistance (STEM), with the exception of location 1 in 2009. Horticultural traits involved with fruit size measurements had higher *R*^2^ values than those associated with fruit quality or plant architecture.

### Means separation

There were significant (*P* ≤ 0.05) differences among genotypic means for all traits, except for CD in location 3 in 2009 (Table S1). In general, sub-NILs with *S. habrochaites* alleles at marker loci TG358 and At1g10500 matured significantly later (DAP1st and/or DAP50) than control cultivar E6203 in at least one trait and year or location combination, and most of these lines also had significantly reduced YLD and 30Wt. Relative to E6203, NIL5 exhibited significantly (*P* ≤ 0.05) greater foliar resistance to *P. infestans* (*i.e.*, lower LEAF values) and increased Brix, but also had later maturity (DAP1st and DAP50) and larger plant size (H, W, SZ). Sub-NILs 08GH5516, 08GH5616, and 08GH5861 also displayed significantly (*P* ≤ 0.05) improved foliar resistance (LEAF) and larger plant size, with the latter two also having significantly higher Brix.

### Correlations

Pearson correlation coefficients (*r*) were obtained for *P. infestans* resistance trait means with horticultural trait means within each year (Table 3). Only significant correlations (*P* ≤ 0.05) ≥0.4 are discussed. CD was weakly negatively correlated with LEAF and STEM (*r* = −0.41; range, −0.42 to −0.46, respectively). HAB was positively correlated with LEAF and STEM (*r* = 0.41; range, 0.57–0.64, respectively), with upright plants having higher AUDPC values (*i.e.*, more susceptible). Maturity traits were negatively correlated with LEAF and STEM only in 2010, although this was influenced by location and time of maturity trait evaluation (DAP1st *vs.* DAP50). Significant (*P* ≤ 0.05) correlations were also found between pairs of horticultural traits (Table S3). Of particular note, YLD was negatively correlated with maturity traits DAP1st and DAP50 in both years (range, −0.50 to −0.78), and CD was significantly positively correlated with the maturity traits in both years (range, 0.53–0.83).

**Table 3:** Trait correlations

2009	LEAF1	LEAF2	
CD4		−0.41^†^	
HAB1234	0.41^†^		
			
**2010**	**LEAF12**	**STEM1**	**STEM2**
			
DAP1st3	−0.53^‡^	−0.44^†^	−0.45^†^
DAP1st4	−0.53^‡^		
DAP50_3		−0.47^†^	
DAP50_4		−0.47^†^	−0.46^†^
YLD3		0.54^‡^	0.46^†^
FH3			0.43^†^
FH4			0.42^†^
FW4			0.45^†^
FP3			0.43^†^
FP4			0.43^†^
30Wt3			0.49^†^
30Wt4			0.50^†^
Brix4		0.45^†^	0.54^‡^
CD34		−0.42^†^	−0.46^†^
HAB34		0.57^‡^	0.64^‡^
H12		0.70^‡^	0.71^‡^
H3	−0.65^‡^		
H4	−0.61^‡^		
W12	0.48^‡^		
W34		0.67^‡^	0.77^‡^

Pearson correlation coefficients (*r*) among *Phytophthora infestans* resistance traits (LEAF and STEM) (data from Johnson *et al.* 2012) and horticultural traits were performed using genotype means. Only significant correlations ≥0.4 are presented. Trait names are given by year according to trait and location(s) (see *Materials and Methods* section).

* *P* ≤ 0.05.

† *P* ≤ 0.01.

‡ *P* ≤ 0.001.

### Linkage mapping

The linkage map for the chromosome 5 introgressed region from *S. habrochaites* was 12.3 cM and spanned markers TG358 to At3g55360 (Figure 1). The average marker spacing was 0.7 cM and the largest gap was 2.5 cM between markers At2g39950 and TG23. Using additional markers developed from the SL2.40 *S. lycopersicum* tomato genome sequence build (http://solgenomics.net), we determined that the introgression in NIL5 extended north of TG358 to at least TG318, and south of At3g55360 to at least CT130. Comparison of our genetic map with the SL2.40 *S. lycopersicum* genome sequence also indicated that the order of markers TG69 and At3g55360 was reversed, suggesting an inversion or possibly errors in the reference genome sequence.

The 12.3 cM region from TG358 to At3g55360 corresponds to a physical distance of 2.35 Mbp in the SL2.40 *S. lycopersicum* genome sequence build, whereas the 12.0 cM region from TG358 to TG69 corresponds to a *S. lycopersicum* physical distance of 2.7 Mbp. However, the physical distances based on *S. lycopersicum* are approximate only because the introgressed region is from *S. habrochaites*, which may have a different physical distance. Based on the physical distance between markers in the *S. lycopersicum* genome sequence, the physical extent of the *S. habrochaites* introgression beyond the boundaries of our linkage map likely includes at least another 896 kb north of TG358 and at least another 680 kb south of TG69.

### Mapped QTL

Within the introgressed chromosome 5 region containing resistance QTL *lb5b* (Brouwer and St. Clair 2004), 67 significant (*P* ≤ 0.05) QTL were detected for 17 horticultural traits (Figure 1 and Table 4). In 2009, 45 QTL were detected; 22 QTL were detected in 2010. If we consider multiple coincident QTL for the same trait as a single, unique QTL, then a total of 41 unique QTL were mapped across the 17 traits.

**Table 4:** Summary of significant QTL for horticultural traits

Trait Class	Trait Code	Trait	Group	Year	Location(s)	Peak Marker or Interval	Peak LOD/Threshold LOD	*R*^2^
Maturity	DAP1st	Time to first ripe fruit (d)	*Hort5-1*	2009	3 & 4	TG358	7.24/1.60	0.42
				2010	3	TG358	7.06/1.68	0.51
				2010	4	TG358	4.82/1.70	0.41
			*Hort5-3*	2009	3 & 4	At2g31970–At4g12590	2.88/1.60	0.16
				2010	3	At2g31970	2.06/1.68	0.11
	DAP50	Time to 50% ripe fruit (d)	*Hort5-1*	2009	3	TG358	4.42/1.71	0.29
				2009	4	TG358	4.90/1.67	0.30
				2010	3	TG358	3.26/1.63	0.30
				2010	4	TG358	6.19/1.79	0.49
Yield	YLD	Yield	*Hort5-1*	2009	3	TG358	13.42/1.63	0.65
				2009	4	TG358	10.09/1.75	0.54
				2010	3	TG358	6.51/1.71	0.49
			*Hort5-3*	2009	4	At2g31970–At4g12590	3.40/1.75	0.16
Fruit size/shape	FH	Fruit height	*Hort5-1*	2009	3	TG358	10.20/1.79	0.42
				2009	4	TG358	5.14/1.68	0.24
				2010	3	TG358	4.49/1.63	0.39
				2010	4	TG358	4.77/1.76	0.41
			*Hort5-3*	2009	3	At2g31970–At4g12590	2.52/1.79	0.07
	FW	Fruit width	*Hort5-2*	2009	3 & 4	At3g17210	4.11/1.64	0.27
	FS	Fruit shape (H:W)	*Hort5-1*	2009	3	TG358	4.97/1.69	0.16
				2009	4	TG358	2.04/1.53	0.05
			*Hort5-2*	2009	3	At5g49510	3.86/1.69	0.12
				2009	4	At5g49510	4.24/1.53	0.12
			*Hort5-4*	2009	4	TG69	2.55/1.53	0.07
	FP	Fruit size	*Hort5-1*	2009	3	TG358	6.83/1.69	0.38
				2009	4	TG358	1.71/1.64	0.13
				2010	3	TG358	2.85/1.74	0.27
				2010	4	TG358	2.25/1.87	0.16
			*Hort5-2*	2009	3	TG23	2.74/1.69	0.12
			*Hort5-4*	2009	3	At4g12590	2.15/1.69	0.09
	30Wt	Fruit weight	*Hort5-1*	2009	3	TG358	7.03/1.79	0.34
				2009	4	TG358	5.09/1.58	0.29
				2010	3	TG358	4.99/1.56	0.36
				2010	4	TG358	4.72/1.80	0.39
			*Hort5-2*	2009	3	TG23	3.85/1.79	0.15
				2009	4	At3g17210	2.07/1.58	0.10
	SW	Seed weight	*Hort5-1*	2009	3 & 4	T0536	2.08/1.60	0.06
Fruit quality	Brix	Brix	*Hort5-1*	2009	4	TG358	4.44/1.60	0.21
			*Hort5-3*	2009	3	At2g31970–At4g12590	7.18/1.74	0.45
				2009	4	At4g12590	6.66/1.60	0.39
			*Hort5-4*	2010	4	T1541–TG69	4.73/1.65	0.41
	pH	pH	*Hort5-1*	2009	3	TG358	2.54/1.68	0.18
Plant architecture	CD	Canopy density	*Hort5-1*	2009	4	TG358	2.31/1.72	0.17
			*Hort5-4*	2009	3	TG69	3.92/1.55	0.27
	HAB	Plant habit	*Hort5-1*	2010	3 & 4	TG358	4.36/1.75	0.38
			*Hort5-2*	2009	1, 2, 3, 4	At5g49510	3.65/1.69	0.25
	H	Plant height	*Hort5-1*	2009	3 & 4	TG358	5.81/1.73	0.32
				2010	1 & 2	TG358	3.68/1.74	0.33
			*Hort5-2*	2009	3 & 4	At2g39950	3.53/1.73	0.19
			*Hort5-3*	2009	1 & 2	At2g31970–At4g12590	4.05/1.73	0.27
				2009	3 & 4	At2g31970–At4g12590	5.32/1.73	0.35
				2010	3	At2g31970	2.96/1.80	0.28
				2010	4	At2g31970	3.78/1.73	0.32
	W	Plant width	*Hort5-1*	2009	1	TG358	3.05/1.65	0.22
			*Hort5-3*	2009	3 & 4	At2g31970–At4g12590	9.28/1.67	0.55
				2010	1 & 2	At2g31970–At4g12590	3.32/1.66	0.30
			*Hort5-4*	2009	2	T1541–TG69	2.17/1.65	0.18
				2010	1 & 2	TG69	3.07/1.66	0.20
	SH	Plant shape (H:W)	*Hort5-1*	2009	3 & 4	TG358	11.19/1.63	0.58
			*Hort5-2*	2009	3 & 4	At3g55800	2.34/1.63	0.08
			*Hort5-3*	2009	1	At4g12590	3.23/1.48	0.22
			*Hort5-4*	2010	3 & 4	TG69	2.11/1.65	0.21
	SZ	Plant size	*Hort5-1*	2009	1 & 2	TG358	3.54/1.73	0.24
				2010	1 & 2	TG358	3.61/1.80	0.27
			*Hort5-2*	2009	3 & 4	At2g39950	2.70/1.62	0.14
				2010	1 & 2	At3g17210	1.88/1.80	0.13
			*Hort5-3*	2009	3 & 4	At2g31970–At4g12590	5.29/1.62	0.35
				2010	3 & 4	At2g31970–At4g12590	2.69/1.67	0.32
**Trait Class**	**Trait Code**	**Trait**	**Group**	**Year**	**Location(s)**	**Allele Direction**	**1-LOD Support Interval**	**Flanking Markers**
Maturity	DAP1st	Time to first ripe fruit (d)	*Hort5-1*	2009	3 & 4	(+)	0.0–0.3	TG358-T0536
				2010	3	(+)	0.0–0.3	TG358-T0536
				2010	4	(+)	0.0–0.3	TG358-T0536
			*Hort5-3*	2009	3 & 4	(+)	7.3–9.9	At5g49510-T1541
				2010	3	(+)	6.3–9.5	At5g49510-T1777
	DAP50	Time to 50% ripe fruit (d)	*Hort5-1*	2009	3	(+)	0.0–0.3	TG358-T0536
				2009	4	(+)	0.0–0.3	TG358-T0536
				2010	3	(+)	0.0–0.3	TG358-T0536
				2010	4	(+)	0.0–0.3	TG358-T0536
Yield	YLD	Yield	*Hort5-1*	2009	3	(−)	0.0–0.3	TG358-T0536
				2009	4	(−)	0.0–0.3	TG358-T0536
				2010	3	(−)	0.0–0.3	TG358-T0536
			*Hort5-3*	2009	4	(−)	6.3–9.9	At5g49510-T1541
Fruit size/shape	FH	Fruit height	*Hort5-1*	2009	3	(−)	0.0–0.3	TG358-T0536
				2009	4	(−)	0.0–0.3	TG358-T0536
				2010	3	(−)	0.0–1.3	TG358-cLEX13G5
				2010	4	(−)	0.0–0.3	TG358-T0536
			*Hort5-3*	2009	3	(−)	7.7–11.9	At2g31970-TG69
	FW	Fruit width	*Hort5-2*	2009	3 & 4	(+)	5.0–6.3	At2g39950-At5g49510
	FS	Fruit shape (H:W)	*Hort5-1*	2009	3	(−)	0.0–0.3	TG358-T0536
				2009	4	(−)	0.0–0.3	TG358-T0536
			*Hort5-2*	2009	3	(−)	5.0–8.7	At2g39950-At4g12590
				2009	4	(−)	5.5–7.6	At2g39950-TG60
			*Hort5-4*	2009	4	(−)	10.9–12.3	T1541-At3g55360
	FP	Fruit size	*Hort5-1*	2009	3	(−)	0.0–0.3	TG358-T0536
				2009	4	(−)	0.0–1.8	TG358-U221402
				2010	3	(−)	0.0–1.3	TG358-cLEX13G5
				2010	4	(−)	0.0–0.3	TG358-T0536
			*Hort5-2*	2009	3	(+)	5.0–6.3	At2g39950-At5g49510
			*Hort5-4*	2009	3	(−)	8.7–11.9	At2g31970-TG69
	30Wt	Fruit weight	*Hort5-1*	2009	3	(−)	0.0–0.3	TG358-T0536
				2009	4	(−)	0.0–0.3	TG358-T0536
				2010	3	(−)	0.0–0.3	TG358-T0536
				2010	4	(−)	0.0–0.3	TG358-T0536
			*Hort5-2*	2009	3	(+)	5.0–6.3	At2g39950-At5g49510
				2009	4	(+)	4.0–7.3	At2g39950-TG60
	SW	Seed weight	*Hort5-1*	2009	3 & 4	(+)	0.0–1.3	TG358-cLEX13G5
Fruit quality	Brix	Brix	*Hort5-1*	2009	4	(+)	0.0–0.3	TG358-T0536
			*Hort5-3*	2009	3	(+)	7.7–9.4	At2g31970-At4g12590
				2009	4	(+)	7.7–9.9	At2g31970-T1541
			*Hort5-4*	2010	4	(+)	9.5–12.3	T1777-At3g55360
	pH	pH	*Hort5-1*	2009	3	(−)	0.0–0.3	TG358-T0536
Plant architecture	CD	Canopy density	*Hort5-1*	2009	4	(+)	0.0–1.3	TG358-cLEX13G5
			*Hort5-4*	2009	3	(−)	9.9–12.3	T1777-At3g55360
	HAB	Plant habit	*Hort5-1*	2010	3 & 4	(−)	0.0–0.3	TG358-T0536
	H	Plant height	*Hort5-1*	2009	3 & 4	(+)	0.0–0.3	TG358-T0536
				2010	1 & 2	(−)	0.0–1.8	TG358-U221402
			*Hort5-2*	2009	3 & 4	(−)	2.7–5.0	At3g55800-TG23
			*Hort5-3*	2009	1 & 2	(+)	7.7–10.9	At2g31970-TG69
				2009	3 & 4	(+)	7.3–9.4	At5g49510-At4g12590
				2010	3	(+)	7.3–9.4	At5g49510-At4g12590
				2010	4	(+)	6.3–9.4	At5g49510-At4g12590
	W	Plant width	*Hort5-1*	2009	1	(−)	0.0–1.3	TG358-T0536
			*Hort5-3*	2009	3 & 4	(+)	7.7–9.4	At2g31970-At4g12590
				2010	1 & 2	(−)	5.9–9.4	At3g17210-At4g12590
			*Hort5-4*	2009	2	(+)	9.9–12.3	T1777-At3g55360
				2010	1 & 2	(+)	12.0–12.3	TG69-At3g55360
	SH	Plant shape (H:W)	*Hort5-1*	2009	3 & 4	(+)	0.0–0.3	TG358-T0536
			*Hort5-2*	2009	3 & 4	(−)	1.8–5.0	U221402-TG23
			*Hort5-3*	2009	1	(+)	7.7–9.9	At2g31970-T1541
			*Hort5-4*	2010	3 & 4	(−)	9.9–12.3	T1777-At3g55360
Plant architecture	SZ	Plant size	*Hort5-1*	2009	1 & 2	(−)	0.0–0.3	TG358-T0536
				2010	1 & 2	(−)	0.0–0.3	TG358-T0536
			*Hort5-2*	2009	3 & 4	(−)	1.8–5.5	U221402-TG23
				2010	1 & 2	(−)	3.0–8.7	At2g39950-At4g12590
			*Hort5-3*	2009	3 & 4	(+)	7.3–9.4	At5g49510-At4g12590
				2010	3 & 4	(+)	6.3–9.4	At5g49510-At4g12590

Group indicates coincident QTL, as defined by colocation of the 1-LOD intervals. *R*^2^ values are the proportion of phenotypic variation explained by the marker–trait association. Allele direction is the direction of the effect of the *S. habrochaites* allele at that QTL, in terms of the trait being measured. The 1-LOD support interval positions refer to the cM distances on the linkage map for the introgressed region from *S. habrochaites*. See Johnson *et al.* (2012) for LEAF and STEM QTL results.

### Horticultural trait QTL groups

Based on their location on the linkage map, four major horticultural trait QTL groups (*Hort5-1* through *Hort5-4*) were delineated (Figure 1 and Table 4) as described in the *Materials and Methods* section. *Hort5-1* contained QTL for every trait evaluated with the sole exception of fruit width (Figure 1 and Table 4). The QTL controlling reproductive traits within this group tended to explain a higher %PV than those of the other groups. This group includes QTL for days to first ripe fruit, days to 50% ripe fruit, yield, fruit height, fruit size, and fruit weight. The only QTL detected for pH and seed weight were also located within this group. QTL in *Hort5-1* also controlled the plant architecture traits canopy density, plant habit, plant height, plant width, plant shape, and plant size (Table 4). There was evidence of genotype × environment interaction (G × E) for plant architecture, because the presence of the *S. habrochaites* allele at these QTL produced denser, taller plants in 2009, whereas it resulted in a smaller, shorter, more prostrate phenotype in 2010. Plants with the wild allele at these QTL also had delayed maturity, reduced fruit size and weight, but slightly higher seed weight and Brix.

The *Hort5-2* QTL group contained QTL for 8 of the 17 horticultural traits evaluated and included primarily QTL for traits involving fruit size, fruit shape, and plant architecture traits (Table 4). The *S. habrochaites* allele at the *Hort5-2* QTL group resulted in a shorter, more prostrate plant architectural phenotype. The wild allele also increased fruit size and weight, primarily as a result of increased fruit width. The *Hort5-3* QTL group contained QTL for 8 of the 17 traits evaluated (Table 4). In contrast to the *Hort5-2* effect on plant size, the *S. habrochaites* allele at the *Hort5-3* QTL group increased plant size (both height and width). Maturity was slightly delayed by the presence of the wild allele in both years, but only in a single location in 2010. The *Hort5-4* QTL group contained QTL for the following 6 of the 17 traits evaluated: fruit shape; fruit size; plant width; plant shape; Brix; and canopy density (Table 4). The *S. habrochaites* allele at the *Hort5-4* QTL group was associated with a wider, more prostrate, and less dense plant architecture and decreased fruit size.

Plant architecture was influenced by the presence of the *S. habrochaites* introgression at each of the four QTL groups, with *Hort5-2* causing a reduction in plant size, *Hort5-3* and *Hort5-4* causing an increase in plant size, and *Hort5-1* having an environmentally dependent effect. Fruit size was impacted by *Hort5-1* and *Hort5-4*, both reducing fruit size, and by *Hort5-2*, which was associated with larger fruit. Delayed maturity and increased Brix were caused by *Hort5-1* and *Hort5-3*, whereas the other two groups had no significant effect on these important traits.

### Horticulture trait QTL and linkage with *P. infestans* resistance QTL

In our companion study (Johnson *et al.* 2012), we detected two and five QTL groups within the introgressed chromosome 5 region controlling foliar (LEAF) and stem (STEM) resistance to *P. infestans*, respectively, with the QTL groups designated as *LFRes5-1*, *LFRes5-2*, and *STRes5-1* through *STRes5-5* (Figure 1). We used markers in common to align the QTL groups and visual inspection. *LFRes5-1* and *STRes5-1* were colocated with *Hort5-1* (Figure 1). *STRes5-2* slightly overlapped *Hort5-2*. *LFRes5-2*, *STRes5-2*, and *STRes5-3* were colocated with *Hort5-3*. *STRes5-4* and *STRes5-5* were colocated with *Hort5-4*.

The *Hort5-1* QTL group included QTL for 16 of the 17 horticultural traits measured. Most of the horticultural trait QTL within this group mapped within marker interval TG358–T0536, and all shared a peak LOD at TG358 (Figure 1 and Table 4). *LFRes5-1* and *STRes5-1* each contained only a single QTL spanning the same interval TG358–T0536, also with peak LOD at TG358, thus the horticultural trait QTL and these resistance QTL were colocated.

QTL were detected in the *Hort5-2* QTL group for 8 of the 17 horticultural traits measured (Figure 1 and Table 4). The 1-LOD support intervals for the QTL within *Hort5-2* and *STRes5-2* overlapped slightly and the peaks for plant height, plant shape, and plant size QTL comprising this group were at At2g39950 and At3g55800. However, *STRes5-2* consisted of only a single QTL with a peak approximately 2.5 cM away at TG23, suggesting that *Hort5-2* is only linked to, rather than colocated with, *STRes5-2*.

The *Hort5-3* QTL group included QTL for 8 of the 17 horticultural traits evaluated, with each QTL having peak LOD either at At2g31970 or in the interval At2g31970–At4g12590 (Figure 1 and Table 4). In contrast, *LFRes5-2* was composed of three overlapping resistance QTL with the common lower boundary marker At4g12590 but with different upper boundaries of markers At3g17210, At2g31970, and At5g49510 (see Table 4 in Johnson *et al.* 2012). The uppermost of these three resistance QTL had peak LOD at At5g49510, whereas the lower two QTL had peaks in the interval At2g31970–At4g12590. Their peak locations suggest a closer linkage of the QTL in *Hort5-3* with the lower two resistance QTL in *LFRes5-2* than with the uppermost QTL. The two QTL for DAP1st and the yield QTL in this group were colocated with *LFRes5-2*, each spanning the interval At5g49510-T1541.

The *Hort 5-4* QTL group included QTL for 6 of the 17 horticultural traits measured, but only for traits related to fruit size, fruit shape, fruit quality, and plant architecture (Figure 1 and Table 4). The colocated resistance QTL groups *STRes5-2* and *STRes5-3* were composed of three QTL spanning interval T1541-At3g55360: one QTL for resistance from the *S. habrochaites* allele with its peak at TG69 and two QTL with opposite allelic effect, with peaks at T1541 and TG69 (see Table 4 in Johnson *et al.* 2012). The horticultural trait QTL and the resistance QTL in this interval exhibited unstable expression across experimental environments, which precluded a more precise determination of the colocalization of these QTL.

### QTL stability and QTL × environment interaction

QTL for yield, fruit size traits (FH, FP, and 30Wt), and maturity traits (DAP1st and DAP50) at *Hort5-1* were detected repeatedly over years and locations, indicating stability of QTL expression (Figure 1 and Table 4). In contrast, the fruit shape QTL in this *Hort5-1* group were detected in two locations in 2009 but not in 2010, suggesting environmental influence on QTL expression. *Hort5-1* also contained QTL for plant height, but depending on the year and location, the wild allele had opposite effects on plant height. In 2009, in Davis, the presence of the wild allele increased height. In 2010, in Salinas, it decreased height. This difference in allelic effect over years was likely attributable to the large environmental differences between the locations and years. Within the region represented by this QTL group, Brix, pH, canopy density, and plant width were only detected in a single location in a single year, indicating QTL × environment (QTL × E) interactions.

The significant QTL at *Hort5-2* were detected only in 2009 in both Davis locations, with the exception of plant size. Therefore, all the QTL in this group exhibited QTL × E interactions.

In general, QTL for architectural traits (plant habit, plant height, and plant size) at *Hort5-3* were stable because they were detected over years and locations. However, differences in allele directionality for plant width indicated QTL × E effects, possibly as a result of different row spacing used in Salinas compared with Davis. Also, a QTL for plant shape was detected only in one location in 2009. The QTL for Brix in this group were detected in both Davis locations, but only during a single year, also suggesting QTL × E interactions.

In group *Hort5-4*, QTL for plant width were detected in one location in 2009 and in two locations in 2010. The other QTL in this region were only detected during a single year (for plant shape and plant size) or in a single location (for fruit shape, fruit perimeter, Brix, and canopy density), again indicating QTL × E interactions and QTL instability.

### Coincidence of QTL between horticultural and resistance traits

The hypergeometric probability distribution was used to test the significance of correspondence of QTL for *P. infestans* resistance (Johnson *et al.* 2012) with those for horticultural traits. The correspondence of LEAF QTL with both DAP1st and yield QTL was significantly different from chance (*P* = 0.047 for each comparison). No other QTL coincidences between *P. infestans* resistance QTL and horticultural trait QTL were significant.

### Selection of sub-NIL breeding lines

The following nine sub-NILs were selected as being potentially useful as breeding lines for development of tomato varieties with improved resistance to *P. infestans*: 08GH5516; 08GH5616; 08GH5861; 08GH6042; 08GH6261; 08GH6288; 08GH6321; 08GH6345; and 08GH6805 (Table S1). We compared these lines with cultivar E6203 to evaluate their potential in breeding. Three lines (08GH5516, 08GH5616, and 08GH5861) had significantly improved foliar resistance (*i.e.*, lower LEAF values) at both Salinas locations in 2010 and had generally lower LEAF values (although not significantly different) in both locations in 2009. Lines 08GH5616 and 08GH5861 had significantly higher Brix in all experiments. Lines 08GH5516, 08GH6288, 08GH6321, and 08GH6805 did not have significantly later maturity and none of the selected lines was significantly taller, except 08GH5616.

## Discussion

### Genetic architecture of horticultural traits

Our higher-resolution mapping of horticultural trait QTL within an introgressed chromosome 5 region from *S. habrochaites* revealed a complex genetic architecture for various traits, including maturity, yield, fruit size, fruit shape, and fruit weight, Brix, canopy density, plant size, and plant shape (Figure 1 and Table 4). Factors contributing to this genetic complexity include QTL previously detected as single QTL fractionating into multiple QTL for a given trait, tight linkages among QTL for multiple traits, the presence of previously unmapped horticultural trait QTL, and QTL with opposite directionality of allelic effects in different environments.

Brouwer and St. Clair (2004) used NILs for the chromosome 5 region introgressed from *S. habrochaites* to map late blight disease resistance and horticultural trait QTL for plant height, plant shape (referred to here as plant habit), maturity, yield, and fruit size. In our present study, the single QTL mapped for each trait in Brouwer and St. Clair (2004) each fractionated into multiple QTL after higher-resolution mapping, a phenomenon reported previously in other mapping studies (Chen and Tanksley 2004; Lecomte *et al.* 2004; Edwards and Mackay 2009; Studer and Doebley 2011; Johnson *et al.* 2012). Studies of tomato interspecific populations have found similarly complex genetic architectures for traits such as fruit size and fruit quality when mapping within defined chromosomal regions (Fridman *et al.* 2002; Yates *et al.* 2004). In contrast, some high-resolution mapping studies reported only single QTL for traits such as Brix (Fridman *et al.* 2000; Fridman *et al.* 2004), fruit shape (Ku *et al.* 2001), and fruit weight (Alpert and Tanksley 1996; Frary *et al.* 2000).

Tight linkages were identified among multiple QTL controlling horticultural traits in our study. The most complex group, *Hort5-1*, contained QTL for 16 of the 17 traits evaluated and all were mapped within a single 1.8 cM interval. Coincident QTL for multiple traits have also been detected within similarly small genetic map distances in other studies of wild species introgressions in cultivated tomato (Frary *et al.* 2003; Yates *et al.* 2004; Gur *et al.* 2010).

Contributing to the genetic complexity of this chromosome 5 region are the presence of QTL for canopy density and plant size, which remained undetected in the previous study by Brouwer and St. Clair (2004). This result may be explained by the presence of closely linked QTL in repulsion with opposite allelic effects on these traits (Figure 1 and Table 4). The individual effects of these QTL can only be separated by mapping with additional recombinants to provide increased resolution (Mackay *et al.* 2009). Higher-resolution mapping has also allowed detection of QTL controlling stem resistance to *P. infestans* (STEM) (Johnson *et al.* 2012) that were previously undetected at lower resolution by Brouwer and St. Clair (2004).

The traits mapped within this region of chromosome 5 from *S. habrochaites* showed diverse genetic complexity, each varying in number of QTL and direction of allelic effects (Figure 1 and Table 4). Yield, maturity (DAP1st), fruit height, and plant habit were each controlled by two QTL with the same direction of allelic effect, whereas fruit width and canopy density were each controlled by two QTL of opposite allelic effect. Fruit shape and Brix were each controlled by three QTL with the same direction of allelic effect, whereas fruit perimeter, plant height, plant width, and plant size were each controlled by three QTL of varying direction of allelic effect. Plant shape had the most complex genetic architecture, being controlled by four QTL with alternating direction of allelic effect. Similar genetic complexity, including multiple closely linked QTL with varying direction of allelic effects, has previously been reported for higher-resolution mapping of QTL in maize (Graham *et al.* 1997), *Drosophila melanogaster* (Pasyukova *et al.* 2000; De Luca *et al.* 2003), mice (Mollah and Ishikawa 2011), and rat (Granhall *et al.* 2006).

Brouwer and St. Clair (2004) originally reported the linkage of *P. infestans* resistance QTL *lb5b* from *S. habrochaites* with QTL controlling plant shape and some reproductive traits. Subsequently, Johnson *et al.* (2012) described fractionation of this single resistance QTL into multiple QTL. In the present study, we used the same set of sub-NILs for chromosome 5 as Johnson *et al.* (2012) and mapped horticultural trait QTL linked to *P. infestans* resistance QTL. Other studies have reported linkage of horticultural trait QTL with disease and pest resistance genes or QTL introgressed from wild tomato species (Tanksley *et al.* 1998; Robert *et al.* 2001). Close linkage of QTL for disease resistance and horticultural traits has been reported for interspecific populations in other crop species, including potato (Visker *et al.* 2003; Danan *et al.* 2011). Similar results have also been observed in intraspecific populations in potato (Bradshaw *et al.* 2004), pepper (Ben Chaim *et al.* 2001; Barchi *et al.* 2007), bean (Miklas *et al.* 2003; Ender and Kelly 2005; Mkwaila *et al.* 2011), and cacao (Brown *et al.* 2007). Horticultural traits associated with resistance QTL may be related causally to the resistance, for example, traits such as plant height, lodging resistance, and canopy density that affect a plant’s ability to avoid environmental conditions conducive to infection. Alternately, the cosegregation of QTL for horticultural traits with resistance traits may be attributable to repressed recombination between loci controlling the two traits, particularly in introgressions from wild species.

### Tight linkage and pleiotropy

Colocation of QTL controlling multiple horticultural traits with each *P. infestans* resistance QTL group (Figure 1) may be attributable either to tight linkage or to pleiotropy (Brown 2002; Chen and Luebberstedt 2010). Our results suggest tight linkage between QTL groups controlling foliar resistance to *P. infestans* (LEAF, designated as LFRes) (Johnson *et al.* 2012) and maturity (Figure 1). This linkage is particularly interesting because of previously reported correlations between these traits and coincidence of maturity and resistance QTL in potato (see *P. infestans Resistance and Plant Maturity* section). Each of the three horticultural QTL groups coincident with *P. infestans* resistance QTL groups (*Hort5-1*, *Hort5-3*, and *Hort5-4*) also contain QTL controlling other (nonmaturity) traits, suggesting tight linkage to resistance QTL (Figure 1). Alternatively, QTL coincidence may be attributable to pleiotropy in some cases, but we cannot determine this from our current data because it would require additional studies involving thousands of segregating progeny (Mackay *et al.* 2009; Chen and Luebberstedt 2010).

Increased mapping resolution in our study revealed that some of the previously identified coincidences among QTL for horticultural and resistance traits in Brouwer and St. Clair (2004) were most likely attributable to tight linkage, rather than to pleiotropy. QTL within *Hort5-2* and *Hort5-4* had 1-LOD support intervals that did not overlap with the two foliar resistance QTL groups *LFRes5-1* and *LFRes5-2* and their coincident stem resistance QTL groups *STRes5-1* and *STRes5-2*. This result suggests that the *Hort5-2* QTL for plant height, plant size, and plant shape are only linked, not pleiotropic, to *LFRes5-1* and *STRes5-1*. Similarly, the *Hort5-2* QTL and the *Hort5-4* QTL for fruit shape, Brix, canopy density, plant width, and plant shape are likely to be linked, not pleiotropic, to *LFRes5-2* and *STRes5-2*. Other studies of coincident QTL controlling different traits in tomato have also found the QTL to be tightly linked, rather than pleiotropic, when mapped at higher resolution using sub-NILs (Monforte *et al.* 2001; Lecomte *et al.* 2004).

### Stability of QTL and QTL × E interaction

The majority of QTL mapped in our study was stably expressed over environments and detected with coincident or overlapping 1-LOD support intervals over multiple years and/or locations. Our inferences regarding QTL stability are limited to two locations across 2 years for all other traits because only the plant architecture traits were evaluated in all four locations (Salinas and Davis). Of the 41 horticultural trait QTL mapped, 29 were stably expressed over multiple environments, including QTL for maturity, yield, fruit size, fruit shape, Brix, plant size and plant shape. In all years and locations, in *Hort5-1* QTL were detected for maturity, yield, fruit height, fruit size, and fruit weight, and in *Hort5-3* QTL were detected for plant height. The remaining 12 horticultural trait QTL were only mapped for a single location within a single year.

Inconsistent detection of QTL across environments (years, locations, and other factors) may be attributable to interaction between expression of the QTL and the environments in which it is evaluated, described as QTL × E (Bernardo 2008; Xu and Crouch 2008; Mackay *et al.* 2009). Such interactions may reduce the efficiency of marker-assisted selection and may ultimately limit the utility of beneficial QTL alleles, depending on the target environments (Xu and Crouch 2008). Traits exhibiting QTL × E in our study included yield, fruit height, fruit shape, fruit size, Brix, pH, canopy density, and plant shape. Whereas many mapping studies conducted across multiple environments report QTL that are stable, QTL instability (*i.e.*, QTL × E) is also common. For example, studies of tomato (Bernacchi *et al.* 1998b; Frary *et al.* 2004), potato (Constanzo *et al.* 2005), and maize (Peng *et al.* 2011) all identified QTL that were stable across multiple environments and others that exhibited QTL × E interactions.

Comparison of our results to those of other QTL mapping studies of tomato was limited because some studies were conducted in only a single environment (Monforte and Tanksley 2000; van der Knaap *et al.* 2002; Semel *et al.* 2006) or did not test for or report on genotype × environment interactions (Causse *et al.* 2007; Mathieu *et al.* 2009). Consequently, we focused our comparisons to studies that mapped QTL in tomato populations evaluated across multiple locations and/or years and that tested for environmental interactions. In these studies, detection of QTL in a single environment was observed for 25–50% (Frary *et al.* 2004; Johnson *et al.* 2012), 10–25% (Bernacchi *et al.* 1998b), and <10% of mapped QTL (Eshed *et al.* 1996; Eshed and Zamir 1996; Tanksley *et al.* 1996; Brouwer and St. Clair 2004; Do *et al.* 2010). Our results (29% of QTL detected in only a single environment) are within the upper range of these studies.

QTL × E may also explain differences in the direction of allelic effects at QTL in some locations compared with others, as exhibited by QTL controlling plant architecture in our study. A QTL in *Hort5-1* increased plant height in both Davis locations in 2009 but decreased plant height in both Salinas locations in 2010, and a similar pattern was observed for plant width QTL in *Hort5-3* (Table 4). Other studies have reported changes in the direction of allelic effect depending on environment, for example, QTL controlling yield, fruit color, and Brix in tomato (Bernacchi *et al.* 1998a), yield in maize (Bouchez *et al.* 2002; Moreau *et al.* 2004), and plant height and kernel weight in wheat (Campbell *et al.* 2003).

### QTL comparisons to previously mapped QTL in tomato and potato

The map resolution of most previously reported QTL are not sufficient for determining precise locations of QTL. Nonetheless, comparisons based on common markers and informed by genomic sequence data suggested correspondence of some of our QTL with previously reported chromosome 5 QTL for disease resistances and horticultural traits in interspecific tomato populations. Bernacchi *et al.* (1998b) reported QTL for yield, soluble solids (Brix), and maturity associated with marker TG69 in lines derived from *S. lycopersicum* × *S. habrochaites*. These QTL may correspond to our *Hort5-3* group QTL for yield, Brix, and days to first ripe fruit, although the low resolution of their map and single marker regression analysis precluded more precise QTL localization. In lines derived from *S. lycopersicum* × *S. pimpinellifolium*, Brewer *et al.* (2007) reported a QTL for heart-shape fruit in the interval TOM152–TG60 that may correspond to QTL we detected for fruit shape in *Hort5-1*, *Hort5-2*, or *Hort5-3*. They also mapped a QTL for distal blockiness (a trait defined by Tomato Analyzer software) in the interval TG60–TG185 that may be coincident to QTL we detected for fruit shape in *Hort5-3* or *Hort5-4*. Using *S. pennellii* introgression lines (ILs), Causse *et al.* (2004) identified a QTL for pH in IL5-3 that may correspond to our *Hort5-1* group QTL for pH and a QTL in IL5-4 for Brix and other sugar-related traits that may be coincident to our *Hort5-3* and/or *Hort5-4* QTL for Brix. With *S. pennellii* ILs, Eshed and Zamir (1996) detected QTL in IL5-4 for plant weight, Brix, and yield that may be coincident to our *Hort5-3* QTL for plant size, Brix, and yield or to our *Hort5-4* QTL for plant size and Brix. Jones *et al.* (2007) used *S. pennellii* ILs to map *SP5G*, a paralog of the *self-pruning* (*sp*) gene for determinant growth, to interval TG351–TG60, which is coincident to the upper portion of our *Hort5-3* group and includes QTL for plant height, plant width, and plant size. The wild allele from *S. pennellii* at the *SP5G* locus delayed the expression of determinacy, which resulted in increased plant height. We observed similarly increased plant height in sub-NILs containing the *S. habrochaites* allele compared to those with the cultivated allele at TG60, which is closely linked to *SP5G*. Semel *et al.* (2006) detected QTL in *S. pennellii* IL5-3 for biomass, Brix, seed weight, and yield that may by coincident with our *Hort5-1*, *Hort5-2*, and/or *Hort5-3* QTL for plant size, plant height, plant width, Brix, seed weight, and yield.

Previous reports of resistance QTL corresponding to the *lb5b* introgressed region are detailed in Johnson *et al.* (2012). Several studies have described QTL for resistance to *P. infestans* on chromosome 5 of potato, a close relative of tomato. The majority of these studies report a resistance QTL located between potato markers GP21 and GP179 (Collins *et al.* 1999; Visker *et al.* 2003; Visker *et al.* 2005; Achenbach *et al.* 2009; Danan *et al.* 2011). This interval coincides with the upper extent of the *S. habrochaites* introgression in NIL5, which suggests that the potato QTL at that interval may correspond to *LFRes5-1* and *STRes5-1*. Some of these studies also reported linkage between the QTL for resistance and QTL for delayed maturity (Collins *et al.* 1999; Visker *et al.* 2003; Visker *et al.* 2005). This maturity QTL in potato may correspond to our maturity QTL at *Hort5-1*.

### *P. infestans* resistance and plant maturity

*P. infestans* resistance (*i.e.*, lower STEM or LEAF values) was significantly negatively correlated with earlier maturity in the sub-NILs (Table 3), indicating that later maturity was associated with increased resistance. As previously noted, resistance QTL groups were also colocated with QTL for maturity traits (Figure 1). The observed QTL correspondence was found to be unlikely because of chance alone, according to a statistical test based on the hypergeometric probability function. A number of studies of the potato have reported a significant positive correlation of increased resistance to *P. infestans* with late maturity (Toxopeus 1958; Collins *et al.* 1999; Bradshaw *et al.* 2004; Danan *et al.* 2009). Linkage of resistance and maturity QTL in potato was noted on chromosome 5, which is syntenic to tomato chromosome 5 (Collins *et al.* 1999; Oberhagemann *et al.* 1999; Visker *et al.* 2003; Bradshaw *et al.* 2004; Visker *et al.* 2005). Overall, our findings agree with the observations of others regarding the correlation of *P. infestans* resistance with maturity and linkage of QTL controlling these traits on chromosome 5.

Alignment of our tomato chromosome 5 map with the potato MetaQTL map (Danan *et al.* 2011) using common markers, facilitated by the Tomato-Expen 2000 map (Fulton *et al.* 2002b) on SGN (http://solgenomics.net), suggests that the potato QTL for resistance and maturity that mapped to marker interval GP21–GP179 likely corresponds to our *LFRes5-1*, *STRes5-1*, and *Hort5-1* QTL groups, respectively. This QTL coincidence suggests conservation of gene function and synteny between these closely related genera for genes controlling these two traits within this region of chromosome 5. Genome-wide conservation of gene function and order between these genera are supported by evolutionary studies of the Solanaceae (Grube *et al.* 2000; Fulton *et al.* 2002a; Wu and Tanksley 2010) and by direct comparisons of the tomato and potato genome sequences (Sato *et al.* 2012).

Late plant maturity may contribute to increased resistance to *P. infestans* because of temporal variation in inoculum production and/or increased susceptibility of plants during the seedling and reproductive growth phases (Collins *et al.* 1999). These factors have primarily been investigated in potato. During an epidemic, *P. infestans* inoculum production increases and then decreases as uninfected host tissue becomes scarce and/or the environment becomes less favorable (Ferrandino 2012). Young plants tend to be more susceptible but exhibit increased resistance during vigorous vegetative growth before becoming more susceptible again during their reproductive phase (Collins *et al.* 1999). In our study, earlier maturing lines may have exhibited greater susceptibility because of the coincidence of favorable environmental conditions for increased *P. infestans* inoculum production with their more susceptible reproductive growth phase. This explanation was supported by our observations that, in both years in the field, symptoms of *P. infestans* infection were first observed while the earlier lines were flowering and setting fruit but the later maturing lines were still growing vigorously. Additional experiments would be required to determine whether the observed correspondence between maturity and resistance had a physiological basis or was merely a coincidence of the planting dates and onset of environmental conditions favorable to pathogenesis.

### Implications for tomato breeding

Our study revealed a complex genetic architecture of QTL for horticultural traits and *P. infestans* resistance within an introgressed region of chromosome 5 from *S. habrochaites*, primarily because of linkage and/or pleiotropy between resistance QTL and horticultural trait QTL and the presence of QTL × E for some traits. This complexity presents challenges for use of wild species QTL alleles in breeding. The beneficial QTL alleles can be useful for tomato breeding if progeny without unfavorable repulsion phase linkages are obtained via recombination. In addition, suitable environments can be targeted for deployment of alleles exhibiting environmental interactions and complementary genetic backgrounds for expression of these alleles can be identified.

We identified favorable progeny sub-NILs that resulted from recombination between linked QTL with alleles in repulsion, for example, at the *Hort5*-3 QTL for Brix (39–45% PV) and the *Hort5-2* QTL for fruit weight (10–15% PV) (Table 4). Based on the genotypes and trait phenotypes of the sub-NILs, the beneficial alleles from *S. habrochaites* at these two horticultural trait QTL and at the *P. infestans* resistance QTL group of largest phenotypic effect (*LFRes5-2*, 18–47% PV) (Johnson *et al.* 2012) are linked in coupling and not pleiotropic to the QTL for which the wild allele has the largest negative effects (%PV) on maturity, yield, fruit height, fruit size, and fruit weight in *Hort5-1* (Figure 1 and Table 4). Colocation of these beneficial QTL alleles linked in coupling makes the marker interval TG23–T1541 a particularly desirable target for marker-assisted breeding. It would be helpful to identify other recombinants with additional favorable allelic combinations at linked QTL, particularly if the resistance QTL *LFRes5-1* and *STRes5-2* are to be used to develop cultivars with improved resistance as well as acceptable horticultural trait phenotypes.

Slightly negative phenotypic effects of linkage drag need not preclude the use of QTL in certain breeding situations. For example, the *Fhb1* QTL for resistance to Fusarium head blight (*F. graminearum*) in wheat has been used in MAS breeding, despite the linkage of this QTL to loci causing a minor delay in heading date (Haeberle *et al.* 2009). Sub-NILs possessing the wild allele at *Hort5-3*, *LFRes5-2*, and *STRes5-2* QTL but lacking the wild parent allele at *Hort5-1*, *Hort5-2*, *LFRes5-1*, and *STRes5-1* QTL, such as sub-NIL 08GH5516 or 08GH6261, may be more immediately useful for breeding improved tomato cultivars because they possess more favorable horticultural trait phenotypes. MAS breeding for target wild alleles at *Hort5-3* simultaneously with background selection against wild alleles at *Hort5-4* in segregating progeny populations from crosses between these donor lines and other cultivated tomato lines would eliminate the negative effects of wild alleles at *Hort5-4*, producing more useful breeding lines.

Breeders prefer to deploy QTL alleles exhibiting stable expression across a wide range of production environments to maximize applicability for crop improvement. However, the presence of QTL × E does not necessarily impede the use of a QTL in breeding for improved cultivars. QTL for specific environments may be useful if those environments happen to be the major target environments for production of that crop (Paterson *et al.* 1991; Asins 2002; Paterson *et al.* 2003). By integrating crop modeling and MAS, QTL with environmental interactions can be exploited in breeding crop ideotypes for particular environments (Yin *et al.* 2003; Cooper *et al.* 2009). The favorable QTL identified in our experiments for Brix and fruit weight in *Hort5-3* were detected in both locations in which they were evaluated, but only during 1 of 2 years. Further assessment of the stability of these QTL effects in target environments for processing tomato production would determine their value for cultivar improvement.

Truncation selection for multiple traits resulted in the selection of the following nine sub-NILs that had improved resistance relative to processing tomato cultivar E6203: 08GH5516; 08GH5616; 08GH5861; 08GH6042; 08GH6261; 08GH6288; 08GH6321; 08GH6345; and 08GH6805 (Table S1). In addition, two (08GH5616 and 08GH5861) had significantly higher Brix than their cultivated parent, E6203, in every environment in which they were evaluated. The selected sub-NILs can be used directly as donor lines for MAS breeding to improve fruit weight, Brix, and *P. infestans* resistance in tomato cultivars (Collard *et al.* 2005; Foolad and Panthee 2012). Additionally, they can be exploited as parents in crosses with other trait QTL donor lines to combine the beneficial effects of QTL alleles for desirable traits via MAS breeding, a technique known as QTL pyramiding (Collard and Mackill 2008; St. Clair 2010; Wang *et al.* 2012). Our most *P. infestans*–resistant sub-NILs with the most acceptable horticultural phenotypes (*e.g.*, 08GH5516 or 08GH6261) could be intercrossed with selected sub-NILs containing late blight disease resistance QTL from chromosome 11 (Johnson *et al.* 2012; J.E. Haggard, E.B. Johnson, and D.A. St. Clair, unpublished results) to breed for improved resistance. Similar efforts could also be pursued for use of beneficial wild alleles at QTL for Brix and fruit weight in breeding for improved horticultural traits in processing tomato.

## Supplementary Material

Supporting Information

Corrigendum
