# Supplementary material for: Linkage Relationships Among Multiple QTL for Horticultural Traits and Late Blight (P. infestans) Resistance on Chromosome 5 Introgressed from Wild Tomato Solanum habrochaites
Source: G3 (Bethesda). 2013 Oct 11;3(12):2131–46. doi: 10.1534/g3.113.007195 (PMC3852376; doi:10.1534/g3.113.007195)
Supplement: Supporting Information [file supp_3_12_2131__index.html]

Linkage Relationships Among Multiple QTL for Horticultural Traits and Late Blight (P. infestans) Resistance on Chromosome 5 Introgressed from Wild Tomato Solanum habrochaites — Supporting Information 

# Linkage Relationships Among Multiple QTL for Horticultural Traits and Late Blight (*P. infestans*) Resistance on Chromosome 5 Introgressed from Wild Tomato *Solanum habrochaites*

## Supporting Information for Haggard, Johnson, and St.Clair, 2013

**Files in this Data Supplement:**

- Supporting Information - File S1 and Tables S1-S3 (PDF, 421 KB)
- File S1 - Methods and markers used for chromosome 5 *Solanum habrochaites* introgression delineation. (PDF, 416 KB)
- Table S1 - Genotypes (sub-NILs and controls), their graphical marker genotypes for the chromosome 5 introgression region, and trait mean separations (.xlsx, 93 KB)
- Table S2 - Raw phenotypic trait data (.xls, 355 KB)
- Table S3 - Correlations for horticultural traits (.xlsx, 22 KB)
