# Supplementary material for: Linkage Relationships Among Multiple QTL for Horticultural Traits and Late Blight (P. infestans) Resistance on Chromosome 5 Introgressed from Wild Tomato Solanum habrochaites
Source: G3 (Bethesda). 2013 Oct 11;3(12):2131–46. doi: 10.1534/g3.113.007195 (PMC3852376; doi:10.1534/g3.113.007195)
Supplement: Supporting Information [file supp_g3.113.007195_FileS1.pdf]

File S1

Methods and markers used for chromosome 5 *Solanum habrochaites* introgression delineation

PCR markers (SCARs) were used to further refine the extent of the *S. habrochaites* chromosome 5 introgression present in NIL5 that contains QTL *lb5b* (Brouwer and St.Clair 2004). Our approach was first to identify overgo sequences from tomato RFLP markers (http://solgenomics.net/) that were previously mapped to the region flanking the TG358-At3g55360 interval, which was the previous extent of the mapped introgression in NIL5. These sequences were then used as queries for BLAST searches of the *S. lycopersicum* SL2.40 genome build to verify their physical location in the *S. lycopersicum* genome. Primers were designed, using Primer3 software, to amplify these sequences in *S. habrochaites* accession LA2099, the BC_1 of *S. lycopersicum* x LA2099 (because DNA from the F_1 hybrid was not available, and BC_1 is a suitable heterozygous equivalent), NIL5, and a representative *S. lycopersicum* control cultivar, E6203. PCR and agarose gel electrophoresis of amplification products were used to identify primer pairs for each marker with amplicons that were polymorphic among the selected genotypes. One SCAR targeted a SNP and was dominant, while the other targeted an INDEL and was codominant.

The reasoning we used for the determination of the extent of the introgression in NIL5 is the following: if a polymorphism exists between LA2099/BC_1 and NIL5/E6203, then the introgression does not extend to that marker, and if a polymorphism exists between E6203 and NIL5/LA2099/BC1, then the introgression must extend to (and very likely beyond) that marker.

Markers used for chromosome 5 *Solanum habrochaites* introgression delineation

Markers are PCR-based SCAR markers. Marker names refer to the original RFLP marker (see SGN website for details). Physical location refers to the location of the forward primer within the *Solanum lycopersicum* SL2.40 genome build. T_m is the PCR annealing temperature in Celsius. Band size is given in base pairs.

Marker	Physical Location	Forward Primer	Reverse Primer	T_m	Band Size, bp	Inheritance
TG318	59915347	CCATAGAAATTGCCGTAAAG	GGAAGCTTATGCTTTGAGTG	55	~250/411	codominant
CT130	64607160	ATGTCTCGGTTCTCCATGTC	CCCACCAGGGTACTTCTTC	57	396	dominant
