# Supplementary material for: Linkage Relationships Among Multiple QTL for Horticultural Traits and Late Blight (P. infestans) Resistance on Chromosome 5 Introgressed from Wild Tomato Solanum habrochaites
Source: G3 (Bethesda). 2013 Oct 11;3(12):2131–46. doi: 10.1534/g3.113.007195 (PMC3852376; doi:10.1534/g3.113.007195)
Supplement: Corrigendum [file supp_3_12_2131_v2_index.html]

Corrigendum 

# Linkage Relationships Among Multiple QTL for Horticultural Traits and Late Blight (*P. infestans*) Resistance on Chromosome 5 Introgressed from Wild Tomato *Solanum habrochaites*

## Corrigendum for Haggard *et al.*, 2013

**Files in this Data Supplement:**

- Corrigendum
