# Supplementary material for: Linkage Relationships Among Multiple QTL for Horticultural Traits and Late Blight (P. infestans) Resistance on Chromosome 5 Introgressed from Wild Tomato Solanum habrochaites
Source: G3 (Bethesda). 2013 Oct 11;3(12):2131–46. doi: 10.1534/g3.113.007195 (PMC3852376; doi:10.1534/g3.113.007195)
Supplement: Corrigendum [file supp_g3.113.007195_Corrigendum_for_Haggard_et_al.pdf]

Corrigendum for Haggard *et al*., *G3: Genes|Genomes|Genetics* 3 (12) 2131-2146.

G3: Genes|Genomes|Genetics, Vol 3, 2131-2146, December 2013, Copyright © 2013 Haggard *et al*.

CORRIGENDUM

In the article by J. E. Haggard, E. B. Johnson, and D. A. St. Clair, (*G3: Genes|Genomes|Genetics* 3: 1143-1149) entitled "Linkage Relationships Among Multiple QTL for Horticultural Traits and Late Blight (*P. infestans*) Resistance on Chromosome 5 Introgressed from Wild Tomato *Solanum habrochaites*" the first header row in Table 4 has been updated to display the correct headings.
